# Supplementary material for: Hypervitaminosis A in type 2 diabetes and its relation with renal function and cardiovascular complications
Source: PLoS One. 2025 Oct 6;20(10):e0333407. doi: 10.1371/journal.pone.0333407 (PMC12500136; doi:10.1371/journal.pone.0333407)
Supplement: Attachment — Submitted filename: Review Comments for PLOS One.docx [file pone.0333407.s001.docx]

**Review Comments for PLOS One**

**Abstract**

Under “Research Design and Method”, please provide statements on the specific study site, including the name of the study site and the sampling technique adopted for this study.

Under “Results” provide figures to support the statement “The HVA prevalence was significantly higher in patients with cardiovascular complications (CVD). Nevertheless, when patients with CVD were stratified according to renal function, the latter emerged as the main factor associated with HVA”. For example what is the proportion and the p-value? These are necessary to appreciate the outcome of this study.

Under “Conclusions”, the state “The main alteration of vitamin A in ambulatory patients affected by type 2 diabetes is hypervitaminosis” should be rephrased for clarity.

**Main Text**

Introduction

The last statement should be moved to the methodology subsection. It is most appropriate to be included in the statements of inclusion and exclusion criteria.

Under “Research Design and Method”, by describing the study site as “our diabetic clinic” does not help international readership. Please state the exact name for the study site.

State the specific duration for the overnight fast.

What constitutes "other biochemical measurements"?

The statement on height and weight measurement should come before body mass index calculation.

Before Statistical Analysis subsection, the authors should provide statements on the sample size determination.

Concerning these statements under “Results”, Vitamin A was determined in 200 ambulatory patients affected by type 2 diabetes. Patients who took vitamin supplements within the six months before the study were excluded. The cut-off levels of vitamin A were ≤ 1.04 and > 2.79 μmol/L for either hypovitaminosis or hypervitaminosis, respectively. These statements cannot be the narratives for the result outcomes. They are better suited for the subsection on methodology.

The authors found that “The sex distribution of HVA was substantially the same, 22.4% women and 32.2% men with a p value of 0.144.” What sampling technique did the authors adopt in arriving at this outcome for the sex distribution?

The authors also suggested that “the levels of VA increased throughout the categories of CKD; G1= 2.26±0.48; G2= 2.40±0.68; G3a= 2.72±0.71; G3b 2.56±0.61 and G4 (only 3 subjects) 3.45±0.79, p for trend <0.001. The increasing level of vitamin A was significant starting from the CKD3a category (post hoc test of Scheffè). These statements of result claimed by the authors in Figure 2 do not exist in the said Figure 2. So it was difficult to verify the outcome.

In the narrative for Table 2, the authors should report the odd ratio, confidence interval and p-values for the association between obesity and HVA.

The subsection “Conclusions” should be rendered “Discussions”

Under “Conclusions”, The statement “Notably, vitamin A concentration began to increase from stage CKD G3a is not supported by the said "Figure 2 ". Please clarify.
